# User Experience of a Semi-Immersive Musical Serious Game to Stimulate Cognitive Functions in Hospitalized Older Patients: Questionnaire Study

**DOI:** 10.2196/57030

**Published:** 2025-01-06

**Authors:** Laurent Samson, Lena Carcreff, Frédéric Noublanche, Sophie Noublanche, Hélène Vermersch-Leiber, Cédric Annweiler

**Affiliations:** 1 Department of Medical and Rehabilitation Care, Angers University Hospital, Angers, France; 2 Research Center on Autonomy and Longevity, Angers Living Lab in Hospital Geriatrics (Allegro), Department of Geriatric Medicine and Memory Clinic, Angers University Hospital, 4 rue Larey, Angers, 49100, France; 3 Laboratory of Psychology of Pays de la Loire, EA 4638, Angers University, Angers, France; 4 Gérontopôle Autonomie Longévité des Pays de la Loire, Nantes, France; 5 Robarts Research Institute, Department of Medical Biophysics, Schulich School of Medicine and Dentistry, The University of Western Ontario, London, ON, Canada

**Keywords:** virtual reality, geriatrics, reminiscence, episodic memory, serious game, neurocognitive disorders, older adults, user experience

## Abstract

**Background:**

Reminiscence therapy through music is a psychosocial intervention with benefits for older patients with neurocognitive disorders. Therapies using virtual or augmented reality are efficient in ecologically assessing, and eventually training, episodic memory in older populations. We designed a semi-immersive musical game called “A Life in Songs,” which invites patients to immerse themselves in a past era through visuals and songs from that time period. The game aspires to become a playful, easy-to-use, and complete tool for the assessment, rehabilitation, and prevention of neurocognitive decline associated with aging.

**Objective:**

This study aimed to assess the user experience (UX) associated with the newly designed serious game.

**Methods:**

After one or several sessions of the game guided by the therapist, patients of the geriatric wards were asked to answer questions selected from 2 widely known UX scales (AttrakDiff and meCUE [modular evaluation of the components of user experience]) with the therapist’s help. The internal consistency of the UX dimensions was assessed through Cronbach α to verify the validity of the dimensions. The level of engagement of the patient throughout the experimental session was also assessed following an internally developed scale, which included 5 levels (interactive, constructive, active, passive, and disengaged behaviors). UX mean scores were computed and presented graphically. Verbal feedbacks were reported to support the quantitative results.

**Results:**

Overall, 60 inpatients with a mean age of 84.2 (SD 5.5) years, the majority of whom were women (41/60, 68%), were included. Their score on the Mini-Mental State Examination (MMSE) ranged between 12 and 29. A majority of patients (27/56, 48%) had no major neurocognitive disorder (MNCD), 22/56 (39%) had mild MNCD, and 7/56 (13%) had moderate MNCD. The results revealed very positive UX with mean values beyond the neutral values for every UX dimension of both scales. The overall mean (SD) judgment was rated 3.92 (SD 0.87) (on a scale of −5 to 5). Internal consistency was acceptable to good for the emotional dimensions of the meCUE. Questionable to unacceptable consistency was found for the other UX dimensions. Participants were mostly active (23/60, 38%) and constructive (21/60, 35%).

**Conclusions:**

These findings demonstrated a very good appreciation of the game by geriatric inpatients. Participants’ and health care professionals’ verbal comments strongly aligned with the quantitative results. The poor internal consistency in the UX dimensions reflected the high heterogeneity among the included patients. Further studies are needed to evaluate the potential benefits of clinical factors such as neurocognitive functions, mood, depression, or quality of life.

## Introduction

Physical frailty and cognitive impairment are common age-related alterations. While the prevalence of cognitive frailty in the community-dwelling older population is low (<5%), it considerably increases in clinical settings [1] and will also increase due to the current demographic transition [2]. Among cognitive impairments, memory loss is one of the earliest signs before the onset of major neurocognitive disorder (MNCD) (ie, dementia) [3]. MNCD, as described by the *Diagnostic and Statistical Manual of Mental Disorders*, *Fifth Edition* (*DSM-5*) [4], refers to a significant decline in cognitive functioning, affecting one or more cognitive domains such as attention, memory, and language. The deficits interfere with the person’s ability to live independently, requiring assistance in daily activities.

Episodic (or autobiographical) memory is defined as the memory of personal life knowledge and personally experienced events [5]. It has a primordial role in personal identity, maintaining the feeling of time continuity [6]. In addition to the mental images that punctuate one’s life, emotion is the privileged mediator of episodic memory. This form of memory is particularly vulnerable to age-linked changes, and its alteration is seen as a hallmark of early mild cognitive impairment and Alzheimer disease [7].

Aside from pharmacological interventions, psychosocial interventions have been developed and adopted with the objective of maintaining or improving the functions, relationships, and well-being of people with cognitive impairments [8]. Among these psychosocial interventions, reminiscence therapy is seen as a credible and efficient intervention. It consists of discussing past events or experiences with the patient, classically with the help of tangible prompts such as pictures, familiar items, music, or sounds [9]. It has been demonstrated in a recent meta-analysis that reminiscence therapy has positive effects on the quality of life, cognition, communication, and mood of people living with MNCD [10].

Virtual reality (VR) and, more recently, augmented reality (AR) (superimposition of VR elements onto the real-world environment) have become popular in the medical field [11]. They were found efficient not only in surgery education and training, but also in pain management, in motor and cognitive rehabilitation after stroke, and in mental health conditions such as anxiety and depression [11]. The advantage of using such supports is to stimulate both cognitive and motor functions [12]. The use of VR and AR has also been investigated in older populations to ecologically assess, and eventually train, episodic memory [7,13], thanks to the high level of immersion. The findings were in favor of positive effects on well-being as well as cognitive function improvements [14,15]. Depending on the immersion level, the technology can be categorized as immersive, semi-immersive, or nonimmersive; semi-immersive being certainly the most appropriate for older patients with cognitive impairments [14]. Nonimmersive VR was found to positively influence the rehabilitation of the most common geriatric syndromes [15].

**Figure 1.**
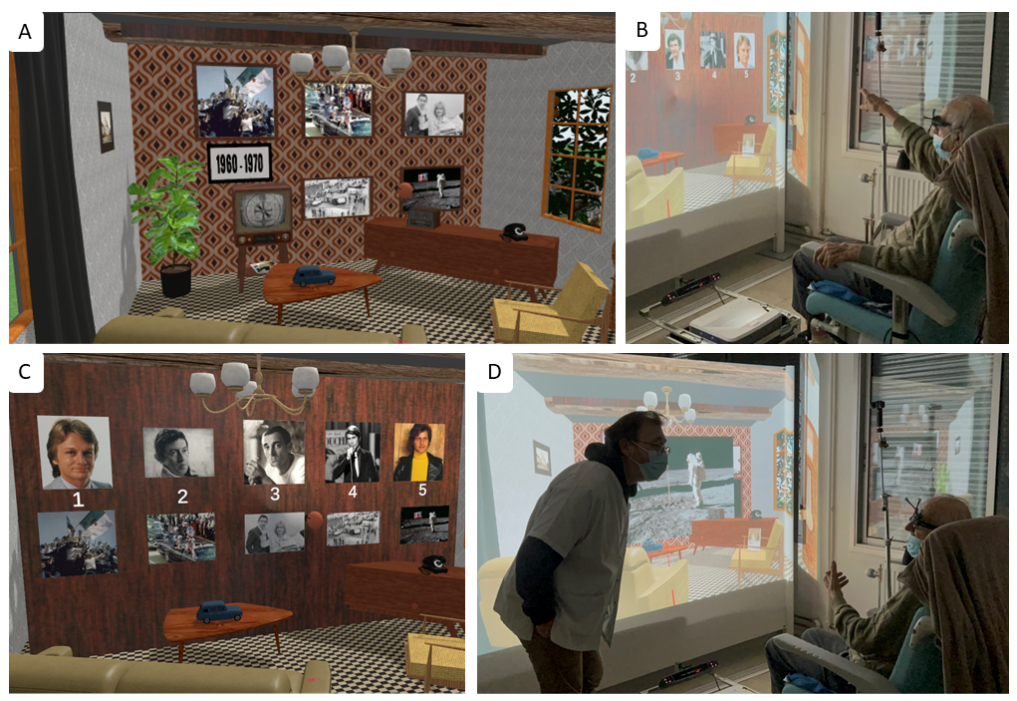
Overview of the game. (A) Virtual living room appearance of the decade 1960‐1970. (B) The virtual reality (VR) device on the floor and the patient wearing VR glasses. (C) Examples of remarkable French singers and events of the decade 1960‐1970. (D) Opened dialogue between the patient and the therapist.

In this context, a semi-immersive musical game called “A Life in Songs” (“*Une vie en Chanson*”) has been designed. It immerses the user in a past decade (from 1950 to 2020), thanks to remarkable songs and events of this decade and thanks to a visual of a decade-related period living room (Figure 1). The game stimulates autobiographical memory without interpretative purpose, in a playful way while allowing to mentally relive autobiographical and semantic events of the user’s life. “A Life in Songs” aspires to become a stand-alone tool for the assessment, rehabilitation, and prevention of neurocognitive decline associated with aging, thus promoting autonomy.

The design of the game required vigilance, in particular regarding ease of use, ergonomics, aesthetics, reliability, and adaptability. A good user experience (UX) is crucial for the success of any digital product. UX has recently emerged in the field of human-computer interaction as an extension of the concept of usability. It helps to consider the whole factors beyond the usefulness of a product, thus considering not only the pragmatic qualities but also the hedonic qualities, the emotions, and the intention to use [16]. This approach has been considered to include humans, their context, and their needs in the creative process. Before conducting a full-scale trial to assess the game’s effectiveness, it is essential to first evaluate its UX.

This study thus aimed to evaluate the UX of the game among a geriatric hospitalized population. A secondary objective was to compare the UX between patients with and without MNCD. We hypothesized that overall UX would be found favorable (above the mean neutral value of the UX scales) and that UX would be similar for both groups of patients.

## Methods

### Study Design

This study was a unicentric nonrandomized cross-sectional UX study using a “task-based experiment” method [17]. It was conducted in the Geriatric Department of Angers University Hospital, supported by the investigative team of its living lab [18], between October 2022 and May 2023.

### Ethical Considerations

The protocol was approved by the ethical committee of Angers University Hospital (2022‐024 and 2023‐038), and it was declared to the National Commission for Information Technology and Civil Liberties (ar21-0159v0). Oral nonopposition to participation was obtained in accordance with French legal requirements for category 3 clinical research [19]. All data were deidentified. No compensation was provided to participants, as the study was integrated into their care program. Consent for publication was obtained from identifiable individuals featured in Figure 1 .

### Description of the Serious Game

“A Life in Songs” (“*Une vie en Chanson*”) is a musical game created in 2017 by LS, the first author of this paper and an art therapist, within the university hospital. The game was initially built on Microsoft PowerPoint support, based on the Goose Game principle where each square represented a year, starting from 1950 until 2020. A remarkable French song and French or international event were associated to each year (square), for instance, *La Javanaise* from Serge Gainsbourg and the assassination of John F Kennedy in 1963. In order to be more attractive and exploitable by other therapists, the game has been redesigned and incorporated into a plug-and-play semi-immersive VR solution (CADWall, Imagin-VR) in 2021. This solution consists of the 3D projection of a virtual living room on a screen wall (Figure 1B). The users wear VR glasses to explore the environment by moving their heads. The game is divided into 7 sections, corresponding to 7 decades (from 1950 to 2020). For each decade, the appearance of the virtual living room changes (decoration and objects according to the period) (Figure 1A). The therapist plays 5 songs from this decade and asks the participant to remember the singer’s name. The 5 corresponding singer faces appear on the virtual wall for help. A total of 5 pictures related to 5 remarkable events of the decade appear on the wall as well (Figure 1C). The game is completely customizable to meet the needs of the patient, therapist, or caregiver. In summary, “A Life in Songs” in VR was designed to immerse patients in the past in an innovative, playful, and pleasant way, aiming to open the dialogue and stimulate episodic memory (Figure 1D). Immersion in the virtual environment promotes natural and instinctive interactions in real time via sensorimotor interfaces. This innovation was also thought to provide health care professionals with an interactive, collaborative, and turnkey tool tailored to their patients and offer the possibility to create their own training protocols. One advantage of the CADWall solution is that it is easily transportable and simple to set up, thus usable in several units of the institution. At the time of the study, the game was not registered with the agency for the protection of programs. It is co-owned by Angers University Hospital and Imagin-VR.

### Recruitment

Patients hospitalized in a geriatric care unit were informed about the study by the medical doctor if they were aged 75 years or older and if they were able to respond to a questionnaire (UX assessment). Interested patients were then approached by the art therapist to give oral and written detailed information about the study and the protocol. Patients under legal protection, as well as non-francophone patients, were not included.

### Protocol

The patients were taken to a specific art therapy room within the geriatric unit by nursing assistants. The patient was allowed to participate on his or her own or accompanied by a relative or with other patients. The material and the game were presented, and the patient was given VR glasses. The investigator (the art therapist) stayed visible to the patient while wearing the glasses. After a few minutes of getting used to the device, the patient was asked to choose a decade to start the game. The investigator launched the corresponding decade: the appropriate living room 3D visual appeared on the virtual wall. A total of 5 songs (from 5 different years of the decade) were played and the patient was asked if he or she knew the singer after each of them. Additionally, after each song, a picture of a remarkable national or international event that happened during the same year appeared on the wall and the patient was asked to talk about this event if he or she remembered it. The investigator facilitated the session in order to guide the patients as much as possible and let them talk about their evoked memories. On the patient’s willing basis, the session could be prolonged by playing with another decade or two.

At the end of the game session, the patient was asked to answer several questions about his or her UX. Finally, the investigator rated the engagement of the patient. Throughout the entire experimental session, the investigator manually recorded verbal feedback. At a later time, health care professionals’ opinions were collected through brief, unrecorded interviews.

### UX Assessment

UX was assessed through 2 self-administered (with the therapist’s assistance if needed) questionnaires inspired from 2 standardized and well-known questionnaires: the AttrakDiff [20] and the modular evaluation of the components of user experience (meCUE) [21] in their French versions [22,23]. The AttrakDiff is composed of 28 items assessing 4 UX dimensions: pragmatic quality (PQ), hedonic quality identification (HQI), hedonic quality stimulation (HQS), and attractiveness (ATT). Each item is presented as a Likert scale semantic differential that represents opposites (eg, “simple-complicated”). The rates range between −3 and 3. To avoid the tendency of acquiescence, the valence of the items was mixed: words on the left of the Likert scale were sometimes positive, sometimes negative [22]. The meCUE is composed of 30 items, structured in 4 modules (product perceptions, emotions, consequences, and global assessment). Each item is presented as a sentence to which the user agrees or disagrees on a Likert scale (from 1 “strongly disagree” to 7 “strongly agree,” except for the item of global assessment which is from −5 “bad” to 5 “good”). The items of the meCUE are categorized into 10 components (usefulness, usability, visual aesthetics, status, commitment, positive and negative emotions, product loyalty, intention to use, and overall evaluation) [24].

Some items have been removed from the standardized questionnaires due to the expected difficulties of understanding the patients with neurocognitive disorders. In total, 12 items were kept from the AttrakDiff (3 of each dimension), and 17 were kept from the meCUE (6 in the module “product perception,” 8 in the module “emotions,” 2 in the module “consequences,” and 1 item of “global assessment”).

### Engagement Behavior

The level of engagement during the whole game session was rated by the experimenter using an internally designed scale called “ICAPD” (interactive, constructive, active, passive, and disengaged), which was inspired from the ICAP (interactive, constructive, active, and passive) hypothesis proposed by Chi et al [25,26]. The ICAPD scale differentiated five engagement behaviors as follows: (1) interactive—the patient discussed, questioned, and debated with the experimenter; (2) constructive—the patient asked questions, constructed his answers, justified them, and offered ideas to the experimenter; (3) active—the patient followed the experimental procedure and answered to the questions; (4) passive—the patient participated summarily in the experimental procedure and answered summarily to the experimenter’s questions; (5) disengaged—the patient did not follow the experimental procedure. The scale is provided in Multimedia Appendix 1. The investigator attributed the level of engagement according to the patient’s dominant behavior during the whole game session.

### Data Collection

The age and sex of the participants, the 29 UX scores, and the engagement levels were collected. The level of neurocognitive disorders assessed through the Mini-Mental State Examination (MMSE) [27] was collected from the computer-based patient record. The MMSE provided a brief screening test that quantitatively assessed the severity of the cognitive disorder. The cutoff value of ≤26 out of 30 was used to dichotomize the cohort into 2 groups: without MNCD (MMSE≥26) and with MNCD (MMSE<26) [28], for the purpose of the secondary objective of this study.

### Data Analysis

The scoring for the items of the AttrakDiff was adapted in order to report negative values for negative valence and positive values for positive valence. In addition, the scoring for the items of the meCUE relative to negative emotions were inverted so that a higher score in this dimension corresponded to fewer negative emotions, and thus better UX. Quantitative variables were presented as mean (SD) or median (IQR), according to the data distribution. Qualitative data were described in terms of numbers and percentages. The UX scores were presented graphically as means and SDs as recommended in a previous study [16]. Comparison of the UX scores between the patients with and without MNCD was performed through Wilcoxon unpaired tests given the low number of participants in each group. Patients with missing MMSE scores were excluded from this analysis. The distribution of each engagement behavior was reported. All descriptive statistics and graphical representations were performed using R (v4.1.1) and the RStudio interface (v1.4) [29]. The internal consistency of the UX dimensions was assessed through Cronbach α when possible (number of items >2 per dimension). Internal consistency was interpreted as excellent if α≥0.9, good if 0.8≤α<0.9, acceptable if 0.7≤α<0.8, questionable if 0.6≤α<0.7, poor if 0.5≤α<0.6, and unacceptable if α<0.5 [30]. The artwork was created in Inkscape.

The analyses and reporting of the results followed the CONSORT-eHEALTH (Consolidated Standards of Reporting Trials of Electronic and Mobile Health Applications and Online Telehealth) guidelines [31].

## Results

**Figure 2.**
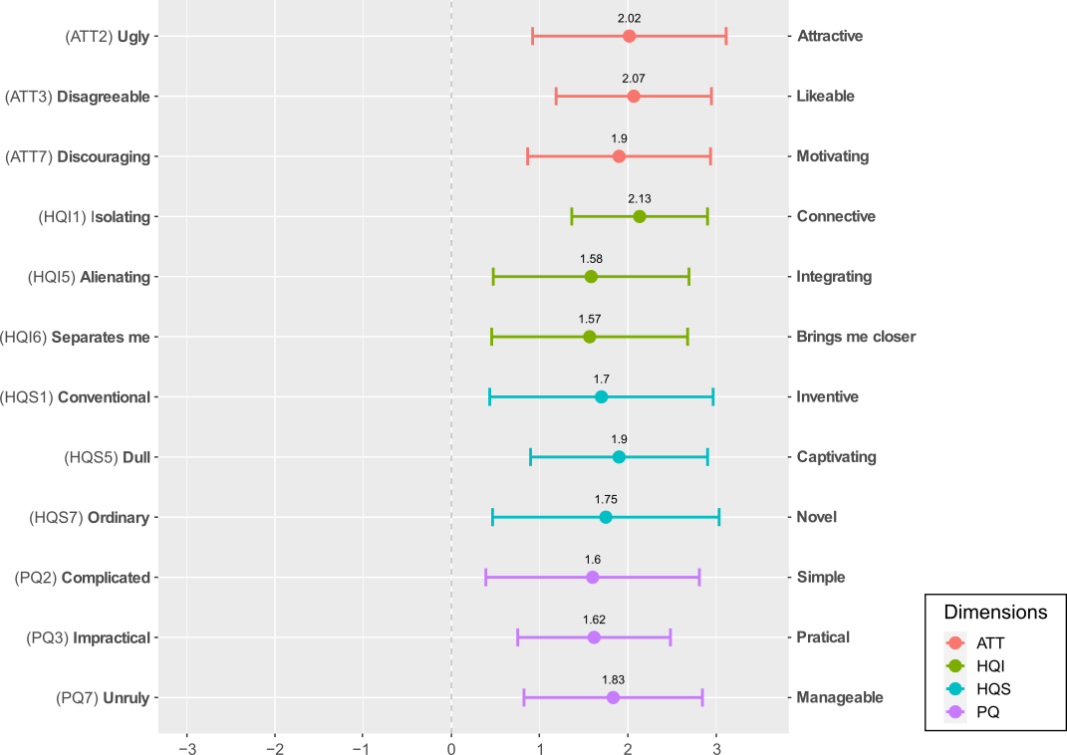
Mean (SD) scores to the AttrakDiff items. ATT: attractiveness; HQI: hedonic quality identification; HQS: hedonic quality stimulation; PQ: pragmatic quality.

**Figure 3.**
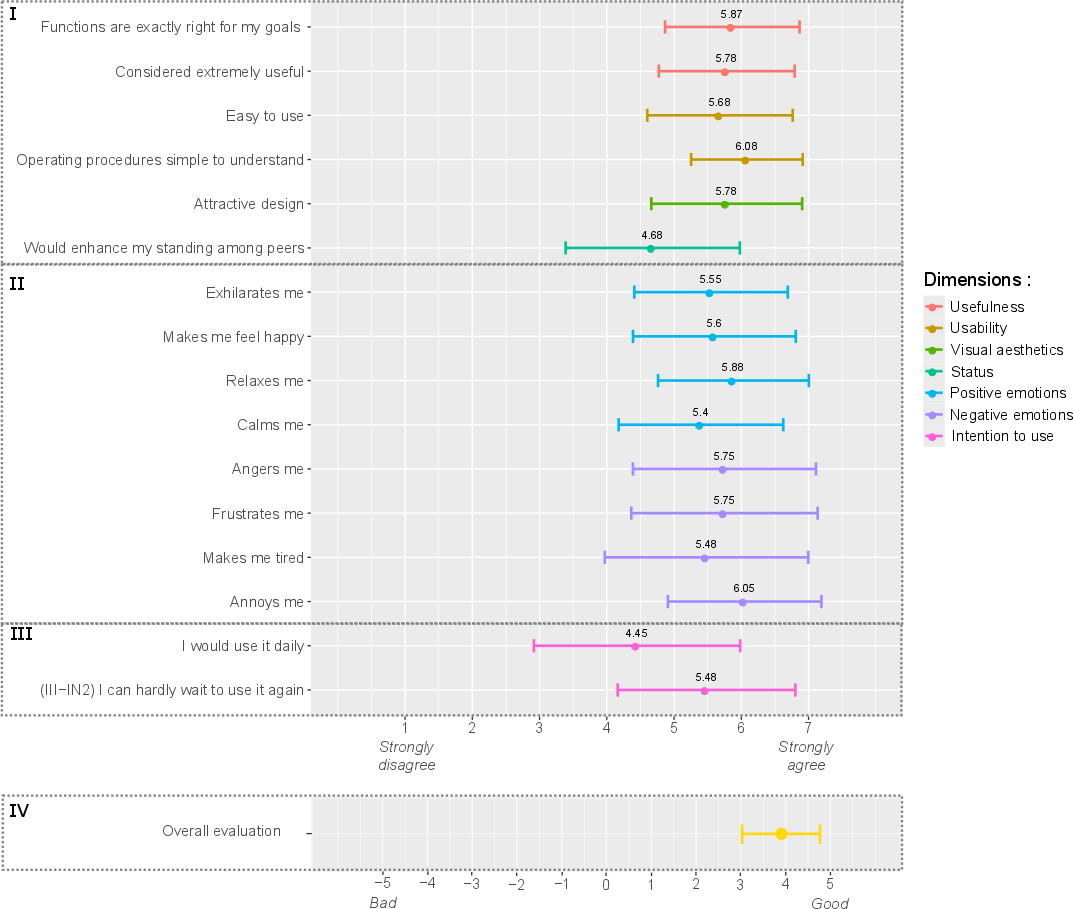
Mean (and SD) scores to the meCUE (modular evaluation of the components of user experience) items: (I) module I: product perceptions; (II) module II: emotions; (III) module III: consequences; (IV) module IV: global assessment.

UX results are presented in Figures 2 and 3. The scores to the AttrakDiff items were all positive (Figure 2) with a mean score of 1.81 (SD 0.2) across all items. The mean (SD) scores obtained for each dimension were as follows: 1.99 (1.01) for ATT, 1.76 (1.04) for HQI, 1.78 (1.19) for HQS, and 1.68 (1.04) for PQ. The scores to modules I, II, and III of the meCUE were also above the neutral value of 4, with a mean score of 5.58 (SD 0.44) across all items of these modules (Figure 3). The lower bounds of the error bars (SDs) for 3 of the 16 meCUE items extended beyond the neutral value 4. Global assessment (module IV) was rated 3.92 (0.87). The details of the scores for each item and each dimension are available in Multimedia Appendix 2.

A total of 60 patients, 41 women and 19 men, with a mean age of 84.5 (SD 5.5) years were included in the study between October 2022 and May 2023. Out of these 60 patients, 4 patients did not have an MMSE score recorded in their computer-based records. Among the 56 patients with available MMSE scores, the mean score was 24.4 (SD 3.9), with a minimum of 12 and a maximum of 29. Using the cutoff value of 26/30, 27 out of 56 (48%) were categorized without MNCD and 29 out of 56 (52%) with MNCD. The main reasons for hospitalization were repeated falls, fractures, memory loss, associated anxiety-depressive disorders, poststroke rehabilitation, Parkinson disease, neurological pathologies, and chronic pain.

**Figure 4.**
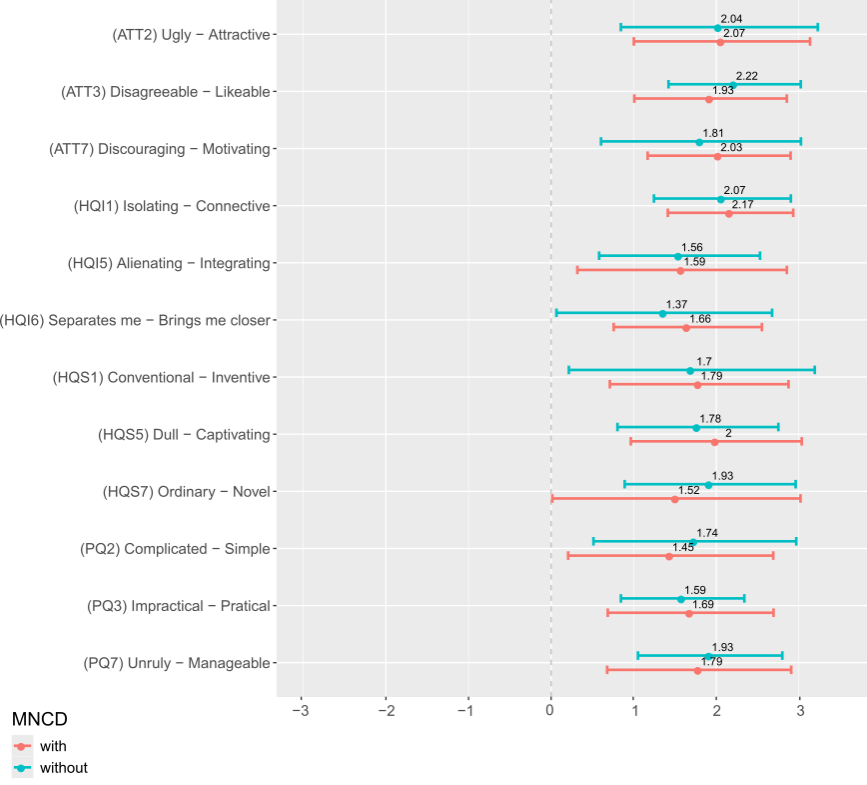
Mean (SD) scores to the AttrakDiff items for the 2 groups of patients with and without major neurocognitive disorder (MNCD). ATT: attractiveness; HQI: hedonic quality identification; HQS: hedonic quality stimulation; PQ:pragmatic quality.

**Figure 5.**
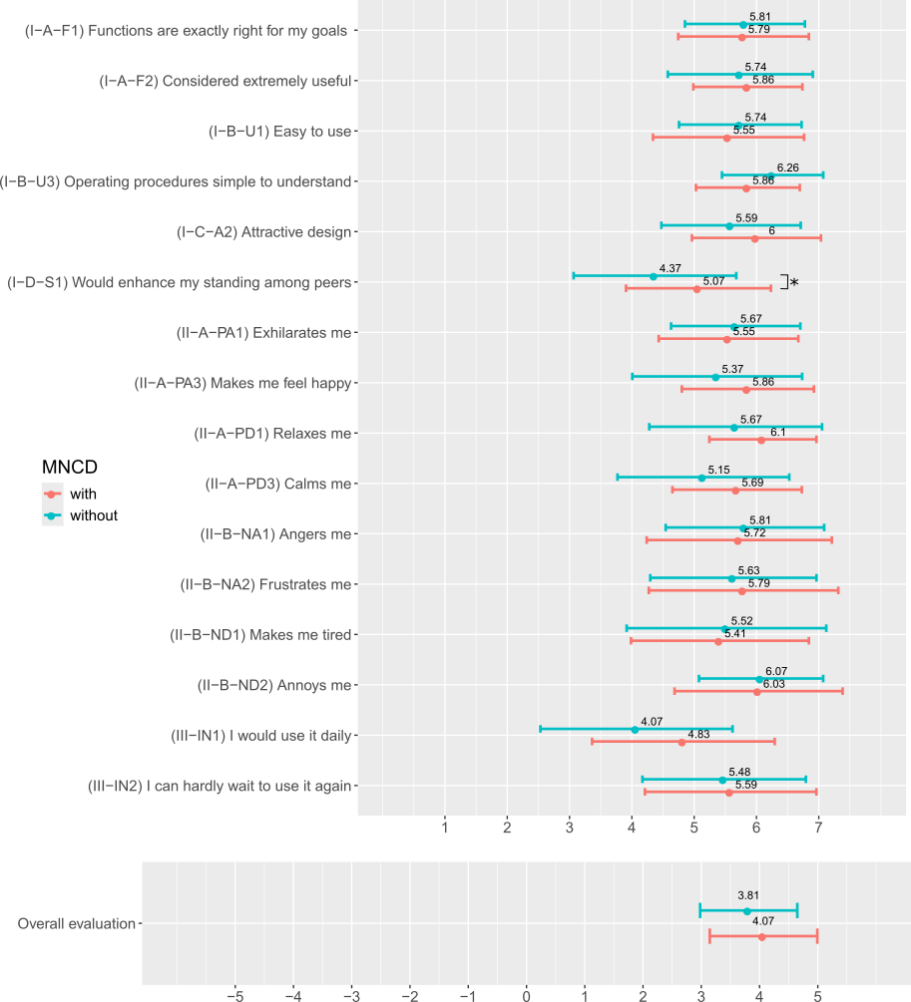
Mean (SD) scores to the meCUE (modular evaluation of the components of user experience) items for the 2 groups of patients with and without major neurocognitive disorder (MNCD). * stands for statistical difference (*P*=.04) between the groups, tested through Wilcoxon unpaired test.

Figures 4 and 5 provide the UX results for the groups of patients with and without MNCD. Among all UX items, no statistical difference was observed between the groups of patients with and without MNCD, with the exception of one item (1/29), the meCUE module I—“The product would enhance my standing among peers” (*P*=.04).

Regarding the levels of engagement, 10 (17%) out of 60 participants were passive, 23 (38%) were active, 21 (35%) were constructive, and 6 (10%) were interactive. None of the participants was rated as disengaged.

Internal consistencies of the UX dimensions assessed are presented in Table 1.

Emotions and perceptions modules showed good and acceptable internal consistency, respectively. ATT, PQ, and consequences modules showed questionable to poor consistency. HQI and HQS showed unacceptable consistency.

**Table 1.** Internal consistency of the assessed user experience dimensions.

		Cronbach α (95% CI)
**AttrakDiff**		
	Pragmatic quality	0.579 (0.256‐0.738)
	Hedonic quality identification	0.411 (0.097‐0.612)
	Hedonic quality stimulation	0.422 (0.011‐0.664)
	Attractiveness	0.608 (0.397‐0.752)
**meCUE^a^**		
	Module I: Perceptions	0.701 (0.541‐0.804)
	Module II: Emotions	0.844 (0.754‐0.899)
	Module III: Consequences	0.655 (0.471‐0.779)

a meCUE: modular evaluation of the components of user experience.

## Discussion

### Principal Findings

This study aimed to assess the UX of geriatric inpatients regarding a semi-immersive musical game designed to stimulate cognitive functions. The findings showed that the game was largely evaluated positively by the targeted population. The patients were mostly actively engaged in the experimentation showing great interest in the game. Patients with and without MNCD appreciated the game similarly.

### Comparison to Prior Work

The instrumental (pragmatic) qualities of the game were well perceived. The patients considered the system useful and usable (meCUE, module I) and simple, practical, and manageable (AttrakDiff, PQ). These results were beyond what could have been expected assuming that the use of VR could be limited due to factors associated with old age [9]. None of the participants complained about discomfort wearing the glasses, nor criticized some aspects of the game such as fluidity, realism, vision difficulties, or cybersickness. In fact, cybersickness and presence were not quantitatively assessed like in studies testing VR in older adults [9,13,32], considering that the game was semi-immersive and the patient saw the therapist guiding the whole session.

It could have been argued that the diversity of support (music, living room visuals, objects, and events) could bring confusion to the patients, especially those with neurocognitive impairment, but this study showed the contrary. Patients acknowledged the variety (“I liked the diversity” [patient 23]). Although most participants indicated they would not play the game daily (meCUE item close to the neutral value), their overall intention to use it was largely positive. The strength of the game was to propose multiple angles of attack to ensure immersion and eventually trigger reminiscence, unlike any other traditional test and therapy [6].

Noninstrumental qualities were also appreciated with good rates on visual aesthetics especially ”meCUE, module I”. While both hedonic qualities were highly recognized, HQIs were particularly highlighted. Notably the connective aspect of the game (AttrakDiff item “HQI1: isolating-connective”) received the highest score. Based on Lallemand et al’s guidelines [16], scores higher than 2 on the AttrakDiff scale can be considered particularly interesting for the product. This was again not expected at this level. This finding demonstrated the success of the game regarding its potential to open the dialogue between the patient and the therapist. A participant commented that the game “is full of life, brings back memories, is cheerful, brings comfort, relaxation and conviviality” [patient 40]. The “status” dimension (item “the product would enhance my standing among peers”) of the meCUE was very close to the neutral value (with the SD extending beyond) and was one of the lowest scores attributed to the game. This could reflect the item being too complex or unsuitable for the population and the product in question.

As reflected by some patients’ comments—“it moved me” [ patient 53] or “it pleased me and did me good” [patient 56], and the scores to the “meCUE, module II”, emotions were also highly positive. The current version of the components of the UX model by Thüring and Mahlke [33] claims that emotions, in addition to perceived product qualities, determine the overall judgment of a product, thus the consequences of use [24]. Our results showed that the overall evaluation (meCUE, module IV) and ATT were closely aligned with the emotions appreciation achieving high scores, and 100% of the participants rated the game positively (minimum rate=2 on a scale of −5 to 5).

From an interviewed psychologist’s point of view, one advantage of the game is to promote the patient’s persistent cognitive capacities without putting him or her in a difficult position. This aspect was also evidenced by patients’ comments (“it makes me realize that I still have memory capacities” [patients 28 and 33]. Game sessions were thus seen as moments of pleasure. This was supported by the results of the AttrakDiff in the ATT dimension, notably with the items “ATT3: disagreeable-likeable” and “ATT7: discouraging-motivating.”

The game was natively designed to suit every patient in geriatric wards. For the purpose of this study, only those capable of answering the UX questionnaires were included. It is worth mentioning that they were all able to play at least with 2 decades of the game, and they perceived it very positively. Their overall judgment (meCUE, module IV) was rated between 3 and 5 (on a scale of −5 to 5). The objective of the game session was not to test the patients’ memory by asking them to name songs or singers but rather to evoke memories and stimulate conversation. This was particularly true for patients with severe neurocognitive impairments (MMSE≤18) [34]. A comparison between patients with lower and higher levels of neurocognitive impairment revealed similar game perceptions. This finding aligns with a larger-scale study involving 313 patients with varying degrees of MNCD, which were enrolled in “task-based experiment”-type UX studies. The conclusions indicated that although engagement may be less active and participation rates lower, patients with severe MNCD were still able to complete the experiments as long as the sessions were properly guided [35].

### Strength and Limitations

Study limitations must be acknowledged. First, nonstandardized sets of UX items were used. The AttrakDiff and meCUE were not used in their complete versions to avoid difficulties with patients presenting neurocognitive disorders. This is why an analysis of internal consistency through Cronbach α tests was carried out. This analysis showed that some dimensions were not consistent (HQI and HQS notably). This is not likely to be attributed to the insufficient number of participants, since the number of participants was higher than usually found in the literature (between 5 and 57 participants) [14]. The absence of using complete questionnaires likely limited the ability to accurately measure certain nuances of the UX and prevented an analysis by dimensions, leading instead to an item-level analysis. Additionally, the ICAPD scale was an innovative method designed to assess engagement. However, since its validity and reliability have not yet been confirmed, this could also be a potential drawback. The ICAPD provided a preliminary estimate of engagement levels, but further validation is needed. In total, the results obtained with the UX and engagement scale may not be comparable with those of other studies using validated methods.

Second, the heterogeneity of the cohort regarding their level of neurocognitive functions should be acknowledged and may have contributed to the low internal consistency of the questionnaires. Detailed results for each item are thus provided in Multimedia Appendix 2, to provide a better insight into the heterogeneity of the results. Third, this study reported verbatim comments heard during the experimental sessions but no proper qualitative study, such as semidirective interviews, was conducted. The investigator did his best to accurately transcribe the verbal comments in order to enrich the present quantitative data. Fourth, cybersickness was not assessed via structured validated scores such as the “virtual reality sickness questionnaire” [36] as recently suggested by Bruno et al [32] for all VR or AR studies. However, it was assessed through spontaneous questions to the participant, and no symptom related to cybersickness was reported.

### Future Directions

Further studies are now needed to assess the impact of the game on clinical aspects. Music therapy or music-based interventions have shown some benefit in people with MNCD [37]. Small short-term benefits on cognitive functions associated with reminiscence therapy were found in a recent Cochrane meta-analysis [10]. The changes in cognitive performance, notably in memory capacity, should thus be assessed when using “A Life in Songs.” It is also possible that not only cognitive function but also quality of life and communication, as well as mood, functioning in daily activities, agitation, and relationship quality, may be improved with the serious musical game [10]. This should not be limited to older populations.

### Conclusions

Because feedback from users is essential in the process of developing new tools, the UX of geriatric inpatients was evaluated regarding the semi-immersive musical game that is aimed to be used for the assessment, rehabilitation, and prevention of neurocognitive disorders in older adults. The 60 patients who tested the musical game in VR reported highly positive UX. Instrumental and noninstrumental qualities as well as emotions were positively perceived, leading to excellent scores of attractiveness and global assessment. Further studies are needed to examine long-term benefits on cognitive functions, mood, and quality of life.

## Supplementary material

10.2196/57030 Multimedia Appendix 1 The internally developed ICAPD (interactive, constructive, active, passive, and disengaged) scale.

10.2196/57030 Multimedia Appendix 2 Detail of the scores obtained for each item of the AttrakDiff and the meCUE (modular evaluation of the components of user experience).

## Abbreviations

AR: augmented reality
ATT: attractiveness
CONSORT-eHEALTH: Consolidated Standards of Reporting Trials of Electronic and Mobile Health Applications and Online Telehealth
HQI: hedonic quality identification
HQS: hedonic quality stimulation
ICAP: interactive, constructive, active, and passive
ICAPD: interactive, constructive, active, passive, and disengaged
meCUE: modular evaluation of the components of user experience
MMSE: Mini-Mental State Examination
MNCD: major neurocognitive disorder
PQ: pragmatic quality
UX: user experience
VR: virtual reality
