# Supplementary material for: User Experience of a Semi-Immersive Musical Serious Game to Stimulate Cognitive Functions in Hospitalized Older Patients: Questionnaire Study
Source: JMIR Serious Games. 2025 Jan 6;13:e57030. doi: 10.2196/57030 (PMC11728198; doi:10.2196/57030)
Supplement: Multimedia Appendix 1 [file games-v13-e57030-s001.docx]

**Appendix 1 – The internally validated ICAPD scale (in French)**

|  | | | | | |
| --- | --- | --- | --- | --- | --- |
| **Grille ICAPD** | | | | | |
| Instructions for the experimenter: As part of using the device, you will observe the patient's behavior. During this single encounter, you are asked to identify the patient's profile. If you observe that the patient exhibits behavior corresponding to one or more of the presented profiles, you will briefly describe the participant's actions using verbs and complements. A list of example actions is provided to assist you in your observations. Some patients may exhibit several profiles, so please specify which one seems the most representative of their dominant profile. | | | | | |
| Profile | **DISENGAGED** | **PASSIVE** | **ACTIVE** | **CONSTRUCTIVE** | **INTERACTIVE** |
|  | Absent | Receptive | Manipulator | Generator | Collaborator |
| General Description of Behavior | The participant does not follow the experimental procedure. | The participant participates minimally in the experimental procedure and gives brief responses to the briefer's prompts. | The participant follows the experimental procedure, uses the tool when possible, and answers the questions. | The participant reorganizes the use of their tool without deviating from the experimental procedure and/or asks questions, constructs their responses, justifies them, and spontaneously proposes ideas to the briefer or in response to the briefer’s questions. | The participant discusses, questions, critiques, reacts, and debates with the caregivers in addition to the briefer. |
| Examples of Participant Actions (non-exhaustive) | Avoiding | Reading | Doing | Asking questions | Participating |
|  | Ignoring | Watching | Describing | Brainstorming | Helping |
|  | Changing the subject | Observing | Breaking down tasks | Commenting | Working in groups |
|  |  | Listening | Activating | Criticizing | Debating |
|  |  |  | Clicking | Comparing |  |
|  |  |  | Deleting | Arguing - Justifying |  |
|  |  |  | Copying | Hypothesizing |  |
|  |  |  | Finding | Suggesting |  |
|  |  |  | Taking notes |  |  |
|  |  |  | Moving |  |  |
|  |  |  | Paraphrasing |  |  |
|  |  |  | Recording |  |  |
|  |  |  | Using |  |  |
| Observations |  |  |  |  |  |
